# Embracing artificial intelligence in nursing: exploring the relationship between artificial intelligence-related attitudes, creative self-efficacy, and clinical reasoning competency among nurses

**DOI:** 10.1186/s12912-025-03306-3

**Published:** 2025-06-20

**Authors:** Amal Diab Ghanem Atalla, Marwa Abd El-Gawad Mousa, Ebtsam Aly Abou Hashish, Naglaa Abdelaziz Mahmoud Elseesy, Aziza Ibrahim Abd El kader Mohamed, Samia Mohamed Sobhi Mohamed

**Affiliations:** 1 Department of Nursing Administration, Faculty of Nursing, Alexandria University, Alexandria, Egypt; 2 Department of Psychiatric and Mental Health Nursing, Faculty of Nursing, Alexandria University, Alexandria, Egypt; 3 Department of Medical-Surgical Nursing, Faculty of Nursing, Badr University, Cairo, Egypt

**Keywords:** Artificial intelligence, AI attitudes, Creative Self-efficacy, Clinical reasoning competency, Nurses

## Abstract

**Background:**

As artificial intelligence (AI) becomes an integral part of healthcare, nursing practice is rapidly evolving, requiring a deeper understanding of how nurses’ attitudes toward AI influence essential competencies such as creative self-efficacy and clinical reasoning competency, both of which are crucial for delivering safe and effective patient care.

**Aim:**

This study aimed to explore the relationship between nurses’ AI-related attitudes, creative self-efficacy, and clinical reasoning competency.

**Methods:**

A cross-sectional descriptive-correlational design was employed, involving a convenience sample of 380 nurses working in critical care units at a university-affiliated hospital in Egypt. Data were collected using three validated instruments: the Nurses’ Artificial Intelligence Attitudes Scale, the Creative Self-Efficacy Scale, and the Clinical Reasoning Competency Scale. Data analysis included both descriptive and inferential statistics.

**Results:**

The majority of nurses demonstrated high levels of AI-related attitudes and clinical reasoning competency, while moderate levels of creative self-efficacy were observed. A strong positive correlation was found between AI attitudes and both creative self-efficacy and clinical reasoning competency (*r* = 0.559 and *r* = 0.728, *p* < 0.001, respectively). Regression analysis confirmed that AI attitudes were significant predictors of both creative self-efficacy and clinical reasoning competency, explaining 37.4% and 56.5% of their variance, respectively. Additionally, educational qualifications and years of nursing experience were identified as significant factors influencing these competencies.

**Conclusion and Implications:**

Positive attitudes toward artificial intelligence (AI) play a crucial role in enhancing nurses’ creative self-efficacy and clinical reasoning competency. Therefore, fostering positive perceptions of AI and providing targeted training are vital to prepare nurses for AI-integrated clinical environments. Integrating AI-focused content into nursing education and promoting continuous professional development are key strategies to strengthen nurses’ readiness to engage with AI-driven healthcare. Additionally, healthcare organizations and nursing leaders should create supportive environments that encourage AI adoption while preserving the principles of patient-centered care.

**Clinical trial number:**

Not applicable.

## Introduction

The rapid advancement of artificial intelligence (AI) is reshaping clinical practice worldwide by enhancing diagnosis, patient care, and decision-making processes. AI technologies, including clinical decision support systems, predictive analytics, and AI-driven monitoring tools, are increasingly adopted in healthcare to improve accuracy, efficiency, and patient outcomes [1, 2]. As AI becomes integral to healthcare delivery, nursing practice must evolve accordingly, requiring not only technical competence but also confidence and readiness to engage with AI tools effectively [3, 4]. Key competencies for navigating AI-enhanced environments include clinical reasoning, which allows nurses to make informed decisions under complex and uncertain conditions, and creative self-efficacy, defined as the belief in one’s capacity to generate innovative solutions to clinical challenges [5]. Nurses’ attitudes toward AI are pivotal in shaping these competencies, as positive attitudes foster motivation, engagement, and openness to adopting new technologies [6]. 

In Egypt, AI holds significant potential to address long-standing challenges in healthcare, such as high patient-to-nurse ratios, workforce shortages, and resource limitations [7]. However, realizing these benefits depends on nurses’ preparedness and willingness to incorporate AI into their clinical practice. Understanding how nurses’ attitudes toward AI influence their clinical reasoning and creative self-efficacy is critical to designing targeted educational and training programs that empower nurses to utilize AI effectively in patient care.

Therefore, this study aims to explore the relationship between nurses’ attitudes toward artificial intelligence (AI), their creative self-efficacy, and clinical reasoning competency. By examining these associations, the study seeks to clarify the factors influencing nurses’ readiness to adopt AI technologies in clinical practice.

## Literature review

### Theoretical underpinning

This study is grounded in three interrelated theories—Bandura’s Social Cognitive Theory (1986) [8], Evans’ Dual Process Theory of Reasoning (2008) [9], and Sweller’s Cognitive Load Theory (1988) [10]—which collectively could explain how AI-related attitudes influence creative self-efficacy and clinical reasoning competency among nurses.

*Social Cognitive Theory* emphasizes that behavior is shaped by the interaction of personal beliefs, environmental factors, and outcomes. Central to this theory is the concept of self-efficacy, which refers to the belief in one’s ability to succeed. Positive attitudes toward AI can enhance nurses’ creative self-efficacy, encouraging them to engage confidently in innovative clinical reasoning [8]. The *Dual Process Theory of Reasoning* distinguishes between intuitive (System 1) and analytical (System 2) thinking. AI can support both reasoning systems by providing timely and predictive data. Nurses’ attitudes toward AI and their creative self-efficacy could influence how effectively they integrate AI-generated insights into clinical decisions [9]. Cognitive load theory focuses on the cognitive effort required for task completion. AI can reduce nurses’ cognitive load by automating routine tasks and analyzing complex data, allowing more cognitive space for creative problem-solving and clinical reasoning. Nurses’ perceptions of AI as a supportive tool depend on their attitudes and self-efficacy [10]. Together, these theories highlight that AI-related attitudes shape both creative self-efficacy and clinical reasoning competency, forming the foundation of this study’s conceptual model.

### Conceptual framework

**Fig. 1:**
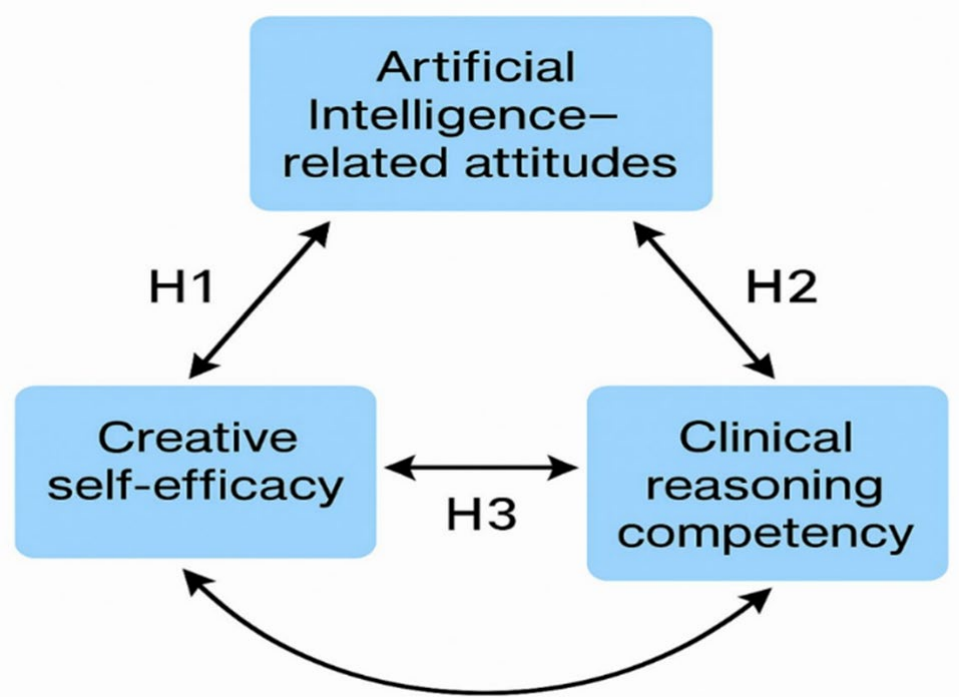
Researchers’ conceptual framework of the study

This study is guided by a conceptual framework that examines the relationships among Artificial Intelligence (AI)-related attitudes, creative self-efficacy, and clinical reasoning competency among nurses. Given the growing integration of AI into healthcare, understanding how nurses perceive AI and how these perceptions relate to their professional competencies is critical for ensuring effective AI adoption in nursing practice (Fig. 1).

### Artificial intelligence (AI) and AI-related attitudes

Artificial intelligence (AI) is increasingly recognized as a transformative technology in healthcare and is applied in nursing to improve patient care, clinical decision-making, and workflow efficiency. AI provides advanced support for clinical decisions, patient monitoring, diagnostics, and administrative tasks [4, 11–13]. One key application is AI-based Clinical Decision Support Systems (CDSS), which analyze patient data in real-time and offer evidence-based recommendations to prevent deterioration, helping nurses make timely, informed decisions and enhancing patient safety [14–16]. AI-driven predictive analytics also identify patients at risk of complications, such as falls or pressure ulcers, by continuously monitoring data and alerting providers for early intervention [15, 16]. AI-enhanced nursing documentation using speech recognition and natural language processing reduces paperwork and allows more focus on direct care [15, 16].

Hence, AI has significant advantages and benefits in nursing, including reducing workload, enhancing diagnostic accuracy, and improving care efficiency. AI supports nurses in making timely, evidence-based decisions and promotes patient safety by minimizing errors and enabling early risk detection [17, 18]. It also facilitates precision nursing by personalizing care based on individual patient needs, ultimately improving care quality and outcomes [17, 18]. Moreover, AI helps streamline documentation and supports clinical tasks, but to ensure its effective and ethical use, appropriate education and training for nurses remain essential [11]. 

*Attitudes toward artificial intelligence* (AI) refer to individuals’ overall perceptions, beliefs, and feelings about AI technologies, including their usefulness, ease of use, trustworthiness, and potential impact on their work and society [11]. In nursing, attitudes toward AI reflect how nurses perceive the integration of AI tools in clinical practice, which can influence their willingness to adopt and use these technologies [19, 20]. Positive attitudes are often associated with greater acceptance and effective use of AI in healthcare settings, while negative attitudes may lead to resistance or fear of AI replacing human roles [21]. Attitudes toward AI encompass cognitive, affective, and behavioral components, reflecting beliefs about AI’s usefulness, trust in AI systems, readiness for adoption, and perceived threats [20]. According to a study by Alruwaili et al. (2024),nurses’ attitudes toward AI significantly affect their readiness to engage with AI-based tools, impacting both workflow and patient outcomes [19]. Positive attitudes toward AI are essential for nurses to embrace AI tools in practice, which can facilitate better clinical reasoning and innovative problem-solving [22]. Despite AI’s potential benefits, nurses’ attitudes toward AI vary, influenced by factors such as limited AI literacy, ethical concerns, fear of job displacement, and doubts about AI reliability [20]. Therefore, examining how AI-related attitudes relate to professional competencies such as creative self-efficacy and clinical reasoning competency is crucial for designing effective training and education programs that prepare nurses for AI-enhanced care environments.

### Creative Self-efficacy

The concept of creative self-efficacy is grounded in Bandura’s Social Cognitive Theory, which posits that self-efficacy beliefs influence motivation, behavior, and performance [8]. Creative self-efficacy is defined as individuals’ belief in their capacity to generate innovative and effective solutions to complex problems [23]. In the nursing context, creative self-efficacy is crucial for adapting to technological advancements, including AI, and for addressing complex patient care needs in creative and patient-centered ways [24]. Nurses with high creative self-efficacy are more likely to engage in problem-solving, adapt AI-generated insights, and innovate care processes [25]. Positive attitudes toward AI are expected to enhance creative self-efficacy by increasing nurses’ confidence in their ability to utilize AI effectively in clinical problem-solving. According to Brockhus et al., creative self-efficacy encompasses both assumptions and beliefs about one’s creativity and evidence-based judgments of creative behavior, reflecting a nurse’s ability to implement innovative care solutions [26].

### Clinical reasoning competency

Clinical reasoning competency refers to the nurse’s ability to systematically assess patient data, analyze problems, make informed decisions, and implement appropriate care plans [27]. Clinical reasoning is a complex cognitive process involving data collection, pattern recognition, diagnostic reasoning, and judgment under uncertainty [28]. It is essential for ensuring safe, patient-centered, and evidence-based nursing care, particularly in dynamic and complex healthcare environments.

AI technologies can support clinical reasoning by providing real-time patient data, risk assessments, and decision support tools, thus enabling nurses to make faster and more accurate clinical decisions [20]. However, the effectiveness of AI in enhancing clinical reasoning depends significantly on nurses’ attitudes toward AI and their creative self-efficacy, that is, their belief in their ability to adapt and creatively integrate AI insights into care [29]. Recent work by Bae et al. outlines three domains of clinical reasoning competency: Self-instruction (the ability to analyze situations independently), intervention strategy regulation (selecting appropriate care interventions), and plan setting (developing comprehensive care plans). These domains align with nurses’ ability to interpret AI-generated data, formulate patient-specific interventions, and adjust care plans dynamically. Therefore, creative self-efficacy may mediate the relationship between AI attitudes and clinical reasoning, as nurses who believe in their creative abilities are better equipped to apply AI insights effectively in clinical practice [30].

#### Study hypotheses

Based on the conceptual framework, this study posits that nurses’ attitudes toward AI are directly associated with both creative self-efficacy and clinical reasoning competency. The following study hypotheses are postulated:

##### H1

There is a significant positive relationship between Artificial Intelligence (AI)-related attitudes and creative self-efficacy among nurses.

##### H2

There is a significant positive relationship between Artificial Intelligence (AI)-related attitudes and clinical reasoning competency among nurses.

##### H3

Creative self-efficacy and clinical reasoning competency among nurses are positively correlated.

#### Significance of study

Although artificial intelligence (AI) is transforming healthcare, limited empirical evidence exists on how nurses’ attitudes toward AI influence essential competencies like creative self-efficacy and clinical reasoning, particularly in developing countries such as Egypt. While nurses recognize AI’s potential, studies show they often lack readiness and confidence in using AI-based tools. Research highlights significant gaps in AI readiness, with studies showing that many nurses lack understanding of AI’s role in clinical settings, contributing to hesitancy in AI adoption [19]. For instance, a study involving 1,243 nurses found that 57% of nurses knew only a little about AI, and 64.7% knew only a little about AI applications in nursing. However, the study also revealed that nurses generally hold positive attitudes toward AI, which may facilitate the dissemination of AI-related knowledge and its application in clinical settings, highlighting the need to understand how attitudes toward AI shape professional capabilities [31]. Moreover, El-Sayed et al. (2025) demonstrated that creative self-efficacy enhances problem-solving and innovation, critical for adapting to AI-supported care, yet the influence of AI attitudes on creative self-efficacy needs more investigation [25].

This study is, therefore, significant as it addresses a critical research gap by examining the relationships among AI-related attitudes, creative self-efficacy, and clinical reasoning competency in Egyptian nursing practice. By providing empirical evidence on how AI perceptions relate to these vital nursing competencies, the study offers insights for healthcare leaders, educators, and policymakers to develop effective training and capacity-building strategies. Understanding these relationships is essential for preparing nurses to adopt AI tools confidently and competently, thereby enhancing patient care quality and safety. Given Egypt’s agenda for healthcare modernization and the global shift toward AI-integrated systems, empowering nurses with the necessary attitudes and skills will be pivotal in improving health outcomes and supporting sustainable healthcare development. Furthermore, the study’s findings may inform broader regional and international efforts in addressing similar challenges in AI integration and workforce readiness.

### Aim of the study

This study aimed to explore the relationship between nurses’ AI-related attitudes, creative self-efficacy, and clinical reasoning competency.

### The research questions are

- What are the levels of artificial intelligence-related attitudes, creative self-efficacy, and clinical reasoning competency skills among nurses?
- What is the relationship between artificial intelligence-related attitudes, creative self-efficacy, and clinical reasoning competency skills among nurses?

## Methods

### Study design and setting

A cross-sectional descriptive design was adopted following STROBE guidelines. The study was conducted at a university-affiliated hospital, which is the largest public teaching hospital, with a capacity of over 6760 beds. This hospital was chosen for its integration of modern technologies, including AI-based tools, and its role in training nurses of various experience levels. All 23 critical care units were included to ensure a broad representation of nurses working in technologically advanced and high-acuity settings, making the results relevant to similar healthcare institutions across Egypt.

### Subjects and sampling

**Fig. 2:**
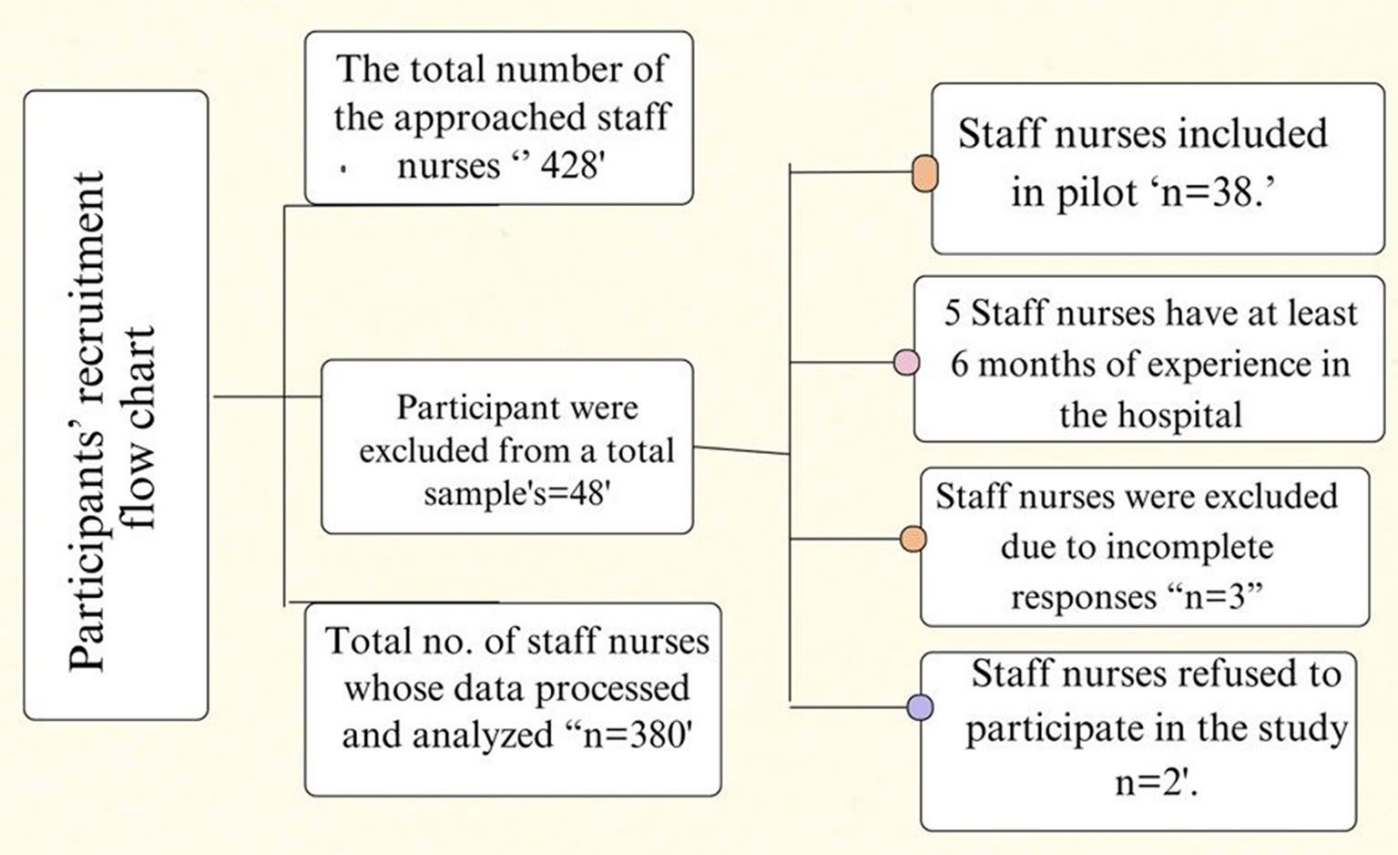
Participants’ recruitment flow chart

The target population comprised 670 staff nurses working in critical care units. Eligible participants were licensed registered nurses with at least six months of continuous work experience in the same department. Nurses on temporary leave or undergoing training during the data collection period were excluded from participation. Using Epi-Info 7, with a 99% confidence level, a 5% margin of error, and a 50% expected frequency, the minimum required sample size was calculated to be 245 nurses. 428 nurses in all were asked to take part in the research. The main analysis did not include the 38 nurses who participated in the pilot research. Out of the 48 potential participants, three provided incomplete responses, two declined to participate, and five nurses had less than six months of experience. As a result, 380 staff nurses made up the final tested sample. (see Fig. 2). It is important to note that the results of this study reflect the experiences of nurses working exclusively in critical care settings. Therefore, caution should be exercised in generalizing these findings to nurses in other healthcare environments.

### Instruments

#### Sociodemographic information questionnaire

This questionnaire was developed by the researchers to gather participants’ background information, including age, gender, educational qualifications, years of nursing experience, and years of experience in their current work unit.

#### General attitudes toward artificial intelligence scale (GAAIS)

Originally developed by Schepman and Rodway (2020) to assess general attitudes toward AI, the GAAIS was adapted and modified by the researchers to suit the nursing context and clinical practice environments [21]. The modifications involved rewording some items to reflect healthcare-specific AI applications (e.g., AI in clinical decision-making and patient care) and adjusting terminology to be relevant to nursing roles. No items were added or removed; rather, the focus of some statements was contextualized to fit nurses’ work settings while retaining the original scale structure and intent. The modified scale contains 20 items, rated on a 5-point Likert scale ranging from 1 (strongly disagree) to 5 (strongly agree). Total scores range from 20 to 100, with higher scores indicating more positive attitudes toward AI. Perception levels are categorized as low (20 ˂46.7), moderate (46.7˂73.4), and high (73.4–100). In this study, Cronbach’s alpha reliability coefficient was 0.90, reflecting excellent internal consistency.

#### Creative Self-Efficacy (CSE) Scale

The Creative Self-Efficacy (CSE) Scale, developed by Brockhus et al. (2014), measures individuals’ confidence in their creative abilities [26]. It includes 15 items, covering two subdomains: assumptions and beliefs about one’s creativity (5 items) and evidence-based judgment of creative behavior (10 items). Responses are rated on a 5-point Likert scale from 1 (strongly disagree) to 5 (strongly agree). Total scores range from 15 to 75, categorized as low (15 ˂ 35), moderate (35 ˂ 55), and high (55–75), with higher scores indicating greater creative self-efficacy. In the current study, Cronbach’s alpha was 0.82, indicating good reliability.

#### Clinical Reasoning Competency Scale (CRCS)

The Clinical Reasoning Competency Scale (CRCS), developed by Bae et al. (2023)was used to assess nurses’ clinical reasoning skills [30]. The scale includes 22 items, divided into three domains: self-instruction (3 items), intervention strategy regulation (8 items), and plan setting (11 items). Responses are measured on a 5-point Likert scale ranging from 1 (strongly disagree) to 5 (strongly agree). Total scores range from 22 to 110, classified as low (22 ˂ 51.3), moderate (51.3 ˂ 80.7), and high (80.7–110). Higher scores reflect stronger clinical reasoning competencies. Cronbach’s alpha in this study was 0.89, demonstrating high reliability.

### Tools validity

All instruments used in this study, including GAAIS, CSE, and CRCS, were translated into Arabic and reviewed in English. A back-translation process was conducted to ensure both linguistic accuracy and conceptual equivalence between the English and Arabic versions of the instrument.

To establish content and face validity, a panel of seven experts in nursing education, clinical practice, and AI-related research evaluated the tools for item clarity, relevance, and cultural appropriateness. Their feedback was carefully incorporated to ensure that the instruments were contextually aligned with the research objectives and suitable for use among the nursing population in Egypt.

To examine construct validity, Confirmatory Factor Analysis (CFA) was conducted using AMOS version 24, with varimax rotation applied to explore the underlying factor structure. The analysis was performed on the final study sample of 363 nurses, exceeding the recommended minimum sample size of 200 participants for factor analysis [32, 33]. Before conducting CFA, Kaiser-Meyer-Olkin (KMO) measures of sampling adequacy and Bartlett’s Test of Sphericity were used to assess data suitability. The KMO values for all instruments exceeded the minimum acceptable threshold of 0.60, with KMO values of 0.918 for CSE, 0.931 for CRCS, and 0.899 for GAAIS, while Bartlett’s Test of Sphericity was significant (*p* = 0.000) for all scales, confirming adequate inter-item correlations.

Based on the theoretical foundations of each scale, factors were extracted for each instrument, and all factor loadings exceeded 0.70, confirming strong item alignment with their corresponding constructs and supporting the tools’ construct validity. Additionally, convergent validity was confirmed as Average Variance Extracted (AVE) values for each dimension exceeded 0.50, indicating that items within each factor shared a high level of common variance [34]. Discriminant validity was verified by comparing the square root of AVE for each construct against inter-construct correlations, ensuring that each construct was empirically distinct.

### Pilot study and reliability

A pilot study was conducted on 10% of the total population (*n* = 38 nurses) to assess the clarity, relevance, and applicability of the instruments, as well as to estimate the time needed for questionnaire completion. The choice of 10% for the pilot study is consistent with established methodological recommendations for survey research [35]. The pilot participants were excluded from the main sample to prevent data contamination. The results of the pilot study confirmed that the tools were clear, appropriate, and practical for use, and no modifications were required.

Furthermore, internal consistency reliability was confirmed through Cronbach’s alpha coefficient for each instrument. In the current study, Cronbach’s alpha was 0.90 for GAAIS, 0.82 for CSE, and 0.89 for CRCS, indicating high reliability. These results are consistent with previous research findings. For instance, Schepman and Rodway originally reported a Cronbach’s alpha of 0.89 for GAAIS [21]. For CSE, Brockhus et al. 2014 reported a Cronbach’s alpha of 0.91, indicating excellent internal consistency [26]. Similarly, CRCS developed by Bae et al. (2023)showed a Cronbach’s alpha of 0.92 in their validation study. Thus, the reliability coefficients obtained in the present study are comparable to and consistent with those reported in the original studies, confirming the reliability of these tools in the Egyptian nursing context [30].

### Overcoming common method Bias (CMB)

To address CMB, the researchers employed a combination of procedural, design, and statistical strategies. Procedurally, participants were assured anonymity and confidentiality, helping to reduce social desirability bias and encourage honest responses. Clear instructions and varied response formats, including reverse-coded items, were used to reduce response patterns. Additionally, different constructs were presented in separate sections of the questionnaire to minimize the risk of common response sets. Statistically, Harman’s single-factor test was conducted, and results indicated that no single factor accounted for the majority of variance, suggesting that CMB was not a significant concern. Furthermore, Confirmatory Factor Analysis (CFA) supported the distinctiveness of the constructs, confirming that the instruments measured separate dimensions. The pilot study also contributed to reducing CMB by identifying and refining any ambiguous or misleading items. By integrating these procedural and statistical techniques, the study effectively minimized the influence of common method bias, thereby enhancing the validity and reliability of the study results.

### Ethical considerations

The study protocol was approved by the Research Ethics Committee of the Faculty of Nursing, Alexandria University (AU-20-8-295). Before participation, the study’s purpose and procedures were fully explained to all nurses, and informed consent was obtained. To ensure confidentiality and anonymity, each questionnaire was assigned a unique code number, and participants were assured that all data collected would be used solely for research purposes. Nurses were also informed of their right to withdraw from the study at any time without any consequences.

### Data collection

Data was collected over three months from August 2024 to October 2024. Each participant received an individualized copy of the questionnaire, which was personally distributed and collected by the researcher. recruitment was conducted through unit managers who distributed the study information and consent forms. Before completing the questionnaire, nurses were given a brief explanation (approximately two minutes) about the study’s aims and instructions. To ensure objectivity and accuracy, nurses were asked to complete the questionnaire in the presence of the researcher, allowing the researcher to address any inquiries and ensure all items were completed properly. The process also enabled effective monitoring of questionnaire distribution and return rates, as the surveys were distributed in specific hospital units. To encourage participation and show appreciation, light snacks were offered to participants. Completing the questionnaire took approximately 15 to 20 min.

### Data analysis

Data were entered and analyzed using IBM SPSS Statistics version 23. Descriptive and inferential statistical analyses were conducted. Normality of the data was assessed using skewness and kurtosis values, and all variables were found to be normally distributed within acceptable ranges. Missing data were minimal; incomplete responses were excluded from the analysis to ensure data integrity.

One-way ANOVA was used to compare mean differences across more than two groups, and Student’s t-test was applied to compare means between two groups for normally distributed quantitative variables. Pearson’s correlation coefficient was used to assess the relationship between AI attitudes, creative self-efficacy, and clinical reasoning competency. Assumptions for t-tests, ANOVA, and regression analyses—such as normality, linearity, homoscedasticity, and independence of errors were checked and met prior to analysis. No specific confounding variables were identified or controlled; however, demographic characteristics were analyzed to explore their associations with study variables. The response rate was 95.5%, with 363 out of 380 distributed questionnaires completed and analyzed. Statistical significance was set at *p* ≤ 0.05 for all tests.

## Results

### Demographic characteristics

Table 1 shows that the majority of nurses (83.9%) were female, between 40 to less than 50 years old (55.3%), with a mean age of 44.07 ± 6.93 years. Additionally, 78.2% of nurses were married, and 51.6% were technical nurses, while only 10% held Bachelor nursing degrees. More than half of the nurses (55.3%) had 5 to less than 10 years of nursing experience, with a mean of 7.85 ± 3.69 years. A similar pattern was observed in their current job positions, as 56.1% had been working in the same position for 5 to less than 10 years, with a mean of 7.14 ± 3.71 years. Moreover, when asked about training courses related to their practice, the vast majority (72.4%) reported not having attended any courses, while only 27.6% had received training.

**Table 1:** Distribution of the studied nurses according to demographic data (*N* = 380)

Demographic characteristics	No.	%
**Sex**		
Male	61	16.1
Female	319	83.9
**Age (years)**		
20–30	5	1.3
30–40	105	27.6
40–50	210	55.3
> 50	60	15.8
Mean ± SD	44.07 ± 6.93	
**Marital status**		
Married	297	78.2
Single	55	14.5
Divorced	5	1.3
Widowed	23	6.1
**Qualification**		
Practical	146	38.4
Technical	196	51.6
Professional (BSN)	38	10.0
**Experience year of nursing**		
1–5	84	22.1
5–10	210	55.3
10–15	76	20.0
More than 15	10	2.6
Mean ± SD	7.85 ± 3.69	
**Experience hospital**		
1–5	81	21.3
5–10	213	56.1
10–15	76	20.0
More than 15	10	2.6
Mean ± SD	7.14 ± 3.71	
**Training course**		
Yes	105	27.6
No	275	72.4

### Descriptive analysis of AI attitudes, creative self-efficacy, and clinical reasoning competency skills

Table 2 reveals the descriptive levels of the studied variables. Regarding AI-related attitudes, nearly two-thirds of the nurses (63.2%) demonstrated high levels, while 31.1% exhibited moderate levels, and only 5.8% had low levels. The overall mean percent score of AI-related attitudes was 67.26 ± 16.08, reflecting a generally positive attitude toward AI.

Overall, creative self-efficacy was moderate among 60.3% of nurses, high among 33.7%, and low among 6.1%, with a mean percent score of 65.0 ± 14.96. Concerning the dimensions of creative self-efficacy, the majority of nurses (79.2%) rated their personal assumptions and beliefs about their own creativity at a moderate level, with a mean percent score of 59.08 ± 19.39. In contrast, the majority (89.2%) perceived their evidence-based assessment of creative self-efficacy as high, with a mean percent score of 70.93 ± 15.17, reflecting a high level of confidence in their creative behavior when evaluated based on practical evidence.

Regarding clinical reasoning competency, 71.8% of nurses scored at a high level, 22.1% at a moderate level, and only 6.1% at a low level, with a mean percent score of 72.10 ± 16.11. Most nurses rated their competency as high in each clinical reasoning dimension, including plan setting (72.9%), intervention strategy regulation (80.5%), and self-instruction (60%).

**Table 2:** Levels and mean percent scores of artificial intelligence- related attitude, creative self-efficacy, and clinical reasoning competency skills (*N* = 380)

	(< 33.3%)		(33.3 – <66.6%)		(≥ 66.6%)		Mean score	Mean percent score
	No.	%	No.	%	No.	%	Mean ± SD	Mean ± SD
**AI- related attitude**	22	5.8	118	31.1	240	63.2	3.69 ± 0.64	67.26 ± 16.08
Personal assumptions/beliefs about own creativity	20	5.3	301	79.2	59	15.5	3.36 ± 0.78	59.08 ± 19.39
Evidence-based assessment of creative self-efficacy	28	7.4	13	3.4	339	89.2	3.84 ± 0.61	70.93 ± 15.17
**Creative self-efficacy**	23	6.1	229	60.3	128	33.7	3.60 ± 0.60	65.0 ± 14.96
Plan setting	23	6.1	80	21.1	277	72.9	4.03 ± 0.68	75.77 ± 17.03
Intervention strategy regulation	28	7.4	46	12.1	306	80.5	3.84 ± 0.68	70.96 ± 16.95
Self-instruction	35	9.2	117	30.8	228	60.0	3.78 ± 0.97	69.58 ± 24.31
**Clinical Reasoning** **Competency**	23	6.1	84	22.1	273	71.8	3.88 ± 0.64	72.10 ± 16.11

### Correlation and linear regression analysis of predictors of nurses’ creative self-efficacy and clinical reasoning competency

The results show that AI-related attitude is positively and strongly correlated with both creative self-efficacy and clinical reasoning skills (*r* = 0.559, *r* = 0.728, *p* < 0.001), respectively. In addition to these positive correlations, Table 3 presents the linear regression analysis examining the predictors of creative self-efficacy and clinical reasoning skills among nurses. Specifically, AI-related attitude significantly predicted creative self-efficacy (B = 0.20, *p* < 0.001), accounting for 37.4% of its variance. Similarly, AI-related attitude significantly predicted clinical reasoning skills (B = 0.47, *p* < 0.001), explaining 56.5% of its variance, indicating a strong predictive effect.

In addition to AI-related attitudes, educational qualifications were a significant predictor for both creative self-efficacy and clinical reasoning skills (B = 2.69, 2.88, *p* < 0.001), respectively. Furthermore, years of nursing experience significantly predicted clinical reasoning skills (B = 3.47, *p* = 0.020) but not creative self-efficacy (*p* > 0.05), indicating that more experienced nurses tend to perceive higher clinical reasoning competencies. Other covariates, including age, hospital experience, and training courses, were not significant predictors for either outcome (*p* > 0.05).

**Table 3:** Linear regression on the effect of creative self-efficacy and clinical reasoning skills among nurses

Predictors	Creative self-efficacy						Clinical reasoning skills							
	B	SE	t	*P*	95% CI		B	SE	t	*p*	95% CI			
					LL	UL					LL	UL		
**Constant**	31.98	2.54	12.57	< 0.001*	26.975	36.980	39.62	3.302	11.99	< 0.001*	33.126	46.110		
**Covariates**														
Age	0.96	0.56	1.71	0.088	-0.143	2.062	1.19	0.728	1.63	0.103	-0.243	2.619		
Qualification	2.69	0.59	4.53	< 0.001*	1.523	3.862	2.88	0.772	3.73	< 0.001*	1.360	4.397		
Experience year of nursing	0.91	1.14	0.79	0.429	-1.340	3.149	3.47	1.482	2.34	0.020*	0.558	6.384		
Experience hospital	0.67	1.12	0.59	0.550	-1.527	2.862	1.41	1.448	0.98	0.330	-4.260	1.436		
Training course	1.08	0.81	1.33	0.185	-2.677	0.520	0.04	1.055	0.04	0.968	-2.033	2.116		
**Main effect**														
**AI-Attitude**	0.20	0.025	8.02	< 0.001*	0.152	0.251	0.47	0.033	14.516	< 0.001*	0.410	0.538		
	*r* = 0.728, R^2^ = 0.374, F = 37.218, *p* < 0.001*						*r* = 0.559, R^2^ = 0.565, F = 80.719, *p* < 0.001*							

R = Pearson correlation, R^2^: Coefficient of determination, B: Unstandardized Coefficients, SE: standard error, t: t-test of significance, LL: Lower limit, UL: Upper Limit, CI: confidence interval, *: Statistically significant at *p* ≤ 0.05

### Nurses’ demographic characteristics and AI attitudes, creative self-efficacy, and clinical reasoning competency skills

As shown in Table 4, several demographic characteristics demonstrated significant differences in perceived AI-related attitudes, creative self-efficacy, and clinical reasoning competency among nurses. Age, educational qualification, years of nursing and hospital experience, and participation in training courses were significantly associated with all three variables (*p* < 0.001 for AI attitudes; *p* = 0.002 for creative self-efficacy; *p* = 0.006 for clinical reasoning competency). Specifically, older nurses, those with higher educational qualifications, greater experience, and those who had attended training reported higher levels across all competencies compared to their peers.

In contrast, sex and marital status showed no significant associations with AI-related attitudes, creative self-efficacy, or clinical reasoning competency (*p* > 0.05), indicating that these factors did not influence nurses’ perceptions or abilities in the studied sample. *(See* Table 4*for detailed values).*

**Table 4:** Perceived difference in AI attitudes, creative self-efficacy, and clinical reasoning competency skills according to nurses’ demographic characteristics

Demographic characteristics	Artificial intelligence attitude		Creative self-efficacy		Clinical Reasoning Competency	
	Mean ± SD.	Test of sig	Mean ± SD.	Test of sig	Mean ± SD.	Test of sig
**Sex**						
Male	72.36 ± 17.80	t = 0.072	56.0 ± 10.15	t = 0.800	86.67 ± 13.90	t = 0.175
Female	72.54 ± 17.31	*p* = 0.942	55.03 ± 8.36	*p* = 0.424	86.34 ± 13.42	*p* = 0.861
**Age (years)**						
20–30	54.33 ± 40.79		42.83 + 14.18		72.50 ± 22.47	
30–40	65.20 ± 22.90	F = 17.326	52.73 ± 10.36	F = 11.445	81.22 ± 18.60	F = 14.459
40–50	76.69 ± 9.96	*p* < 0.001*	56.95 ± 6.90	*p* < 0.001*	89.57 ± 7.52	*p* < 0.001*
> 50	78.14 ± 9.44		56.28 ± 6.09		89.53 ± 7.98	
**Marital status**						
Married	73.38 ± 17.09		55.28 ± 8.47		86.20 ± 13.37	
Single	69.73 ± 18.59	F = 1.216	54.80 ± 7.93	F = 0.287	87.56 ± 12.58	F = 0.478
Divorced	65.80 ± 15.55	*P* = 0.304	52.0 ± 12.86	*P* = 0.835	80.80 ± 21.91	*P* = 0.698
Widowed	69.39 ± 18.01		55.57 ± 11.77		87.30 ± 15.42	
**Qualification**						
Practical	66.35 ± 23.05	F = 19.884	52.12 ± 9.76	F = 31.622	80.49 ± 17.13	F = 28.466
Technical	75.09 ± 10.08	*p* < 0.001*	55.86 ± 6.27	*p* < 0.001*	89.28 ± 8.19	*p* < 0.001*
Professional	82.84 ± 13.29		63.50 ± 8.81		94.21 ± 10.63	
**Experience year of nursing**						
1–5	58.99 ± 27.60		49.48 ± 11.55		73.60 ± 20.06	
5–10	73.23 ± 7.63	F = 38.337	55.81 ± 5.98	F = 24.025	88.94 ± 6.54	F = 46.143
10–15	83.71 ± 11.94	*p* < 0.001*	58.53 ± 7.78	*p* < 0.001*	92.62 ± 9.60	*p* < 0.001*
More than 15	85.80 ± 12.51		64.70 ± 9.18		93.20 ± 10.92	
**Experience hospital**						
1–5	59.32 ± 28.12		49.36 ± 11.38		75.10 ± 20.67	
5–10	73.61 ± 8.76	F = 30.761	55.89 ± 6.28	F = 21.099	88.46 ± 8.07	F = 31.691
10–15	82.59 ± 12.08	*p* < 0.001*	58.74 ± 8.19	*p* < 0.001*	92.11 ± 9.15	*p* < 0.001*
More than 15	79.20 ± 11.91		60.50 ± 8.15		90.60 ± 8.68	
**Training course**						
Yes	78.12 ± 11.35	t = 4.902	57.49 ± 8.86	t = 3.167	89.47 ± 9.50	t = 2.769
No	70.36 ± 18.75	*P* < 0.001*	54.31 ± 8.44	*p* = 0.002*	85.22 ± 14.56	*p* = 0.006*

F: One way ANOVA test t: Student t-test *: Statistically significant at *p* ≤ 0.05

## Discussion

The integration of artificial intelligence (AI) into healthcare has transformed nursing by enhancing diagnosis, patient care, and decision-making. Effective AI adoption requires not only technical skills but also positive attitudes, which influence nurses’ confidence in clinical reasoning and creative problem-solving both essential for high-quality, patient-centered care [2, 3]. This study explores how AI-related attitudes are associated with these key competencies among nurses within the Egyptian healthcare context.

### Descriptive levels of AI attitudes, creative Self-Efficacy, and clinical reasoning competency skills

The findings revealed that the majority of nurses exhibited high and positive attitudes toward artificial intelligence (AI). This suggests that nurses may feel more confident and optimistic as AI becomes increasingly recognized as a technology that enhances healthcare efficiency, accuracy, and clinical decision-making. Such results may be attributed to the perceived benefits of AI in clinical settings. It is well-established that AI technologies facilitate healthcare delivery by streamlining workflows, improving productivity, offering personalized treatment options, supporting patient monitoring and follow-up, and reducing healthcare costs [11, 36]. Positive perceptions among nurses may also be shaped by AI’s capacity to alleviate workload, automate repetitive tasks, and ultimately improve patient outcomes. Additionally, exposure to AI through hospital-based systems or training programs may contribute to heightened trust and enthusiasm toward AI applications in nursing practice. Particularly in Egypt, where healthcare systems often struggle with resource constraints, nurses may perceive AI as a valuable tool for overcoming systemic barriers and enhancing the quality of patient care [37].

This result is in line with Kaplan and Uçar (2023), who reported that nurses held highly positive attitudes toward AI in clinical settings [38]. Similarly, Atalla et al. (2023) noted that nurses demonstrated moderate levels of positive attitudes toward AI, indicating varying levels of acceptance depending on exposure and experience [39]. Furthermore, a study by Ghazy et al. (2023) found that the majority of nurses (58.4%) expressed a good attitude toward AI use in healthcare, while 41.6% expressed negative attitudes, reflecting ongoing variability in AI acceptance within nursing populations [40]. These findings highlight the growing openness among nurses to adopt AI in their professional roles, although continuous education and supportive environments remain essential to ensure the effective integration of AI technologies into nursing practice.

Moreover, there was a notable proportion of nurses who exhibited moderate levels of creative self-efficacy, particularly regarding their assumptions and beliefs about their creative capacities. This suggests that while nurses value creativity, they prefer to apply it within evidence-based frameworks to ensure safe and effective care. Such reliance on evidence-based procedures reflects a balance between innovation and clinical safety, indicating a preference to innovate within validated clinical standards. This finding could be attributed to the nature of nursing practice, which emphasizes patient safety, adherence to clinical protocols, and evidence-based care [13]. It also reflects a desire to combine innovation with proven clinical recommendations to optimize patient outcomes [29]. 

Additionally, this tendency might be influenced by clinical exposure and structured environments that stress evidence-based decision-making, enhancing nurses’ confidence to apply creativity within scientifically grounded frameworks. Supporting this view, Xiang et al. and Liu et al. found that nurses’ creative self-efficacy was strongly linked to their behavior in implementing patient safety improvements [29, 41]. Similarly, Ebrahim et al. revealed that more than half of the studied nurses demonstrated moderate levels of creative self-efficacy and innovative behaviors, reflecting comparable trends [42]. However, the moderate level of creative self-efficacy observed in this study highlights the need for educational strategies and targeted interventions that foster creative thinking while reinforcing evidence-based practices, enabling nurses to develop innovative yet safe clinical practices and patient care.

Furthermore, the majority of nurses in this study demonstrated a high level of overall clinical reasoning competency, including its key dimensions: plan setting, intervention strategy regulation, and self-instruction. This result may be attributed to the recognized importance of clinical reasoning and critical thinking as essential competencies for providing high-quality care and making effective clinical decisions in complex healthcare settings. Nurses may also appreciate their critical role in assessing patient conditions, identifying needs, and delivering timely interventions to ensure patient safety and optimal outcomes. These competencies are often shaped and strengthened through clinical experience and practice.

These findings are supported by Esteron et al. (2025) and Mohammadi et al. (2024), who emphasized that clinical reasoning is a fundamental aspect of nursing practice, encompassing patient assessment, problem analysis, intervention planning, and reflective learning. Mastering these skills not only enhances care quality but also prepares nurses to navigate clinical complexity and take on leadership roles [43, 44]. Furthermore, Lee et al. (2016) confirmed that nurses consistently apply clinical reasoning across all steps of the nursing process [45], while Abd Elshafy (2024) reported that more than two-thirds of nurse educators rated clinical reasoning among nurses as strong, reinforcing its importance as a core nursing competency [46].

### Correlation and linear regression between AI-related attitudes and each of creative self-efficacy and clinical reasoning competency skills among nurses

The current study revealed strong positive correlations among AI-related attitudes, creative self-efficacy, and clinical reasoning competency skills among nurses. Regression analysis further confirmed that AI-related attitude is a significant positive predictor of both creative self-efficacy and clinical reasoning competency, explaining 37.4% of the variance in creative self-efficacy and 56.5% in clinical reasoning competency, indicating a strong predictive effect. These findings suggest that nurses with positive attitudes toward AI are more likely to engage creatively in clinical problem-solving and to apply effective clinical reasoning strategies. This relationship reflects the interconnected nature of technology acceptance, creativity, and critical thinking in modern nursing practice. Positive perceptions of AI may foster openness to technological solutions, encouraging nurses to integrate AI tools into care processes and thus enhancing their ability to analyze complex clinical cases effectively [11, 12]. 

These results align with existing literature highlighting the interconnectedness of technological acceptance, creativity, and critical thinking in nursing practice. Positive attitudes toward AI have been consistently associated with greater readiness to adopt AI technologies and to innovate in clinical care. For instance, Tuncer and Tuncer (2024) found that nurses with positive attitudes toward AI were more inclined to integrate AI tools into their clinical practice, potentially enhancing their creative problem-solving abilities and adaptability to complex care situations [47]. Similarly, Alruwaili et al. (2024) demonstrated that nurses who perceived AI favorably were more likely to embrace innovative approaches and technologies, contributing to enhanced clinical reasoning and decision-making processes [19].

Moreover, the positive correlation between AI-related attitudes and creative self-efficacy observed in this study suggests that nurses who perceive AI positively may be more inclined to leverage these technologies to enhance their creativity in clinical practice. Similarly, several studies have shown that creative self-efficacy is directly linked to innovative behaviors in clinical settings. For instance, Adil et al. demonstrated that nurses with higher creative self-efficacy were more proactive in introducing new technologies and innovative practices in their workplaces [48]. In line with this, Xiang et al. (2023) found that nurses’ creative self-efficacy was strongly associated with their ability to implement innovative and safe patient care interventions [29]. Likewise, Liu et al. (2022) highlighted that creative self-efficacy contributes to innovative behaviors through the enhancement of nursing information competencies, which are crucial for adopting AI-driven solutions in clinical care [41]. Together, these studies emphasize that nurturing creative self-efficacy, along with fostering positive AI attitudes, can enhance nurses’ innovative problem-solving abilities, promote flexible clinical decision-making, and improve the quality and safety of patient care.

Furthermore, the significant predictive relationship between AI-related attitudes and clinical reasoning competency underscores the potential of AI to enhance evidence-based nursing practice and support clinical decision-making. Nurses who maintain positive attitudes toward AI may be more inclined to use AI-based tools to gather, analyze, and interpret clinical data, leading to more accurate and timely decisions in patient care. This is consistent with Kwak et al. (2022), who reported that nurses with favorable AI perceptions demonstrated improved clinical decision-making processes and higher self-efficacy, enabling them to approach patient care with greater confidence and competence [49]. Similarly, Nilsen et al. emphasized that the integration of AI technologies in healthcare is a promising strategy for improving clinical reasoning and decision-making, particularly in complex care situations where data-driven insights are critical [50]. These findings align with Morales-García et al. (2025) who highlighted that nurses’ positive attitudes toward AI significantly influence their job performance, reinforcing the notion that AI readiness is a crucial component of modern nursing competencies. This may reflect that when nurses trust AI tools, they are more confident in making informed decisions, leading to better patient outcomes [51].

### Perceived difference in AI-related attitudes, creative self-efficacy, and clinical reasoning competency skills according to nurses’ demographic characteristics

The study found that nurses’ perceived AI-related attitudes, creative self-efficacy, and clinical reasoning competency were significantly correlated with age, educational background, years of nursing experience, and training program participation.” This revision enhances readability and appropriately captures the relationships found in the data. Older nurses and those with 10 to 15 years of nursing and hospital experience showed higher scores in clinical reasoning and AI attitudes compared to younger or less experienced nurses. Moreover, nurses with professional qualifications scored highest in all areas, and those who attended training programs demonstrated higher clinical reasoning and more positive AI-related attitudes, reflecting the role of advanced education in shaping professional competencies. These findings may be explained by the fact that older and more experienced nurses have greater clinical exposure and experience in critical decision-making, which enhance their reasoning abilities and openness to using AI. Additionally, nurses with professional qualifications are more likely to have been trained in evidence-based practice, critical thinking, and emerging healthcare technologies, including AI, giving them a stronger foundation for both creative and reasoning skills. In contrast, sex and marital status were not significantly associated with AI attitudes, creative self-efficacy, or clinical reasoning skills. This suggests that these competencies and attitudes are shaped more by professional factors (such as education and experience) rather than personal demographic attributes like gender or marital status. This is consistent with other studies indicating that professional development and exposure play a more critical role in shaping nurses’ attitudes and abilities than gender-based or marital factors.

This result is consistent with Attalla et al. (2024), who found that nurses aged between 30 and 40 demonstrated the highest mean scores in AI perception and attitudes [39]. Similarly, Al-Sabawy reported significant variations in AI perceptions based on educational degree levels, indicating that education plays a crucial role in shaping attitudes toward AI [52]. Supporting this, Alruwaili et al. highlighted those nurses with higher education levels showed greater readiness to adopt AI technologies and demonstrated better clinical problem-solving abilities [19]. Furthermore, Liu et al. confirmed that nurses’ information processing and clinical reasoning competencies are significantly influenced by their educational background and years of professional experience, enabling them to better adapt to AI-integrated healthcare environments [41]. These findings align with broader research emphasizing that higher education and practical experience are essential for developing advanced nursing competencies necessary for modern, technology-driven healthcare settings.

In contrast, Alrasheeday et al. (2023) and Elsyed et al. (2020) found that younger nurses tend to hold more positive attitudes toward technology compared to older nurses, which supports the present study’s findings regarding age-related differences in AI attitudes [53, 54]. Similarly, Alruwaili et al. (2024) reported that gender and years of nursing experience did not significantly influence AI attitudes, confirming that experience alone may not be sufficient to shape nurses’ perceptions of AI [19]. These findings may reflect generational differences among nurses, as emphasized by Hwang et al. (2024), who noted that nurses from different generations vary in how they perceive and adapt to technological innovations. Therefore, designing tailored educational and administrative interventions that consider generational differences is essential to effectively prepare nurses for AI integration in healthcare settings [55].

## Conclusion

This study highlights that nurses’ attitudes toward AI significantly influence their creative self-efficacy and clinical reasoning competencies, both critical for safe and effective patient care. Positive AI attitudes are linked to stronger clinical decision-making and innovative problem-solving skills. Furthermore, higher educational qualifications and professional experience contribute to enhancing these competencies, emphasizing the value of academic and clinical preparation. To improve AI readiness, ongoing education, professional development, and tailored training programs are essential. Ultimately, fostering positive AI perceptions and continuous learning will enable nurses to confidently navigate AI-integrated healthcare environments, advancing nursing practice and improving patient care quality.

## Strengths and limitations

A key strength of this study is its focus on nurses’ AI readiness in Egypt, a country undergoing significant healthcare transformation. By addressing this specific context, the study provides valuable insights that can inform regional and local nursing practices as AI technologies are introduced. Additionally, the use of a large and diverse sample can enhance the generalizability and reliability of the findings. However, the cross-sectional design of the study limits the ability to draw causal inferences about the relationships among AI attitudes, creative self-efficacy, and clinical reasoning. Furthermore, the data collection setting may have influenced participants’ responses and potentially affected the authenticity of self-reported data. The use of self-reported data may introduce response bias, as nurses might overestimate or underestimate their AI-related competencies or attitudes, as well as their clinical reasoning and creative self-efficacy. Future longitudinal and experimental studies are recommended to explore these relationships over time and validate the findings in different nursing and healthcare contexts.

### Implications of the study

#### Implications for nursing practice

The findings highlight the importance of fostering positive attitudes toward AI to enhance nurses’ clinical reasoning and creative self-efficacy. AI has the potential to improve clinical decision-making and care delivery; however, its successful integration depends on nurses’ preparedness and confidence in using these technologies. Creating supportive work environments where nurses are encouraged to adopt AI can strengthen their clinical judgment and problem-solving abilities, ultimately improving patient outcomes. Furthermore, providing accessible training and resources will enable nurses to effectively incorporate AI into daily practice, supporting safe and efficient care.

#### Implications for nursing management

Nurse leaders play a critical role in facilitating AI adoption by promoting a culture of learning and innovation. They should ensure that nurses have ongoing access to AI-focused education, practical training, and AI tools. Additionally, nurse managers should work to integrate AI competencies into clinical standards and policies, while maintaining a focus on human-centered care. By encouraging interdisciplinary collaboration and open communication, nurse leaders can ease the transition to AI-enhanced environments and build nurses’ confidence in using AI ethically and effectively.

#### Implications for nursing education and policy

Nursing education must prioritize the inclusion of AI-related competencies in curricula, preparing nurses for increasingly digital healthcare environments. This should not only cover technical skills but also develop the mindset needed to embrace AI as a supportive tool. Educational programs should emphasize ethical considerations, critical thinking, and creative problem-solving in AI-supported care. Policymakers should promote ongoing professional development programs on AI integration, ensuring that nurses across different experience levels are equipped to function in AI-driven settings. Furthermore, policies should support AI innovation in nursing while safeguarding patient-centered practices and ethical standards.

#### Implications for future research

Future research should explore the long-term impact of AI use on nurses’ clinical reasoning and creative self-efficacy, examining how these competencies evolve with sustained AI exposure. Longitudinal studies are essential to understand how attitudes and skills change over time as AI becomes a routine part of nursing care. Additionally, research should investigate the influence of organizational factors, such as leadership support, workplace culture, and training initiatives, on nurses’ willingness and ability to adopt AI.

## Data Availability

The datasets generated and analyzed during the current study are available from the corresponding author upon reasonable request.

## Abbreviations

AI: Artificial Intelligence
AI-Attitude: Artificial Intelligence-related Attitudes
CSE: Creative Self-Efficacy
CRCS: Clinical Reasoning Competency Scale
CDSS: Clinical Decision Support Systems
SPSS: Statistical Package for the Social Sciences
CFA: Confirmatory Factor Analysis
KMO: Kaiser-Meyer-Olkin Measure of Sampling Adequacy
AVE: Average Variance Extracted
SD: Standard Deviation
ANOVA: Analysis of Variance
IBM SPSS: International Business Machines Statistical Package for the Social Sciences
